# Hair-Growth-Promoting Effect of Conditioned Medium of High Integrin α_6_ and Low CD 71 (α_6_^bri^/CD71^dim^) Positive Keratinocyte Cells

**DOI:** 10.3390/ijms16034379

**Published:** 2015-02-19

**Authors:** Chong Hyun Won, Yun-Mi Jeong, Sangjin Kang, Tae-Sung Koo, So-Hyun Park, Ki-Young Park, Young-Kwan Sung, Jong-Hyuk Sung

**Affiliations:** 1 Department of Dermatology, Asan Medical Center, University of Ulsan College of Medicine, Seoul 138-736, Korea; E-Mail: chwon98@chol.com; 2 Department of Applied Bioscience, CHA University, Seoul 135-081, Korea; E-Mails: iteps951@gmail.com (Y.M.J.); sjkang@cha.ac.kr (S.K.); 3 Graduate School of New Drug Discovery and Development, Chungnam National University, Daejeon 305-764, Korea; E-Mail: kootae@cnu.ac.kr; 4 Coway Cosmetics R&D Center, Seoul 153-792, Korea; E-Mail: shpark@coway.co.kr; 5 Asan Institute for Life Sciences, Seoul 138-736, Korea; E-Mail: piaoid05@naver.com; 6 Department of Immunology, School of Medicine, Kyungpook National University, Daegu 700-422, Korea; E-Mail: ysung@knu.ac.kr; 7 College of Pharmacy, Yonsei University, Incheon 406-840, Korea

**Keywords:** conditioned media, hair growth, keratinocyte stem cells, paracrine effect

## Abstract

Keratinocyte stem/progenitor cells (KSCs) reside in the bulge region of the hair follicles and may be involved in hair growth. Hair follicle dermal papilla cells (HFDPCs) and outer root sheath (ORS) cells were treated with conditioned medium (CM) of KSCs. Moreover, the effects of KSC-CM on hair growth were examined *ex vivo* and *in vivo*. A human growth factor chip array and RT-PCR were employed to identify enriched proteins in KSC-CM as compared with CM from keratinocytes. KSC-CM significantly increased the proliferation of HFDPCs and ORS cells, and increased the S-phase of the cell cycle in HFDPCs. KSC-CM led to the phosphorylation of ATK and ERK1/2 in both cell types. After subcutaneous injection of KSC-CM in C3H/HeN mice, a significant increase in hair growth and increased proliferation of hair matrix keratinocytes *ex vivo* was observed. We identified six proteins enriched in KSC-CM (amphiregulin, insulin-like growth factor binding protein-2, insulin-like growth factor binding protein-5, granulocyte macrophage-colony stimulating factor, Platelet-derived growth factor-AA, and vascular endothelial growth factor). A growth-factor cocktail that contains these six recombinant growth factors significantly increased the proliferation of HFDPCs and ORS cells and enhanced the hair growth of mouse models. These results collectively indicate that KSC-CM has the potential to increase hair growth via the proliferative capacity of HFDPCs and ORS cells.

## 1. Introduction

The hair follicle (HF) is a unique characteristic of mammals; its structure undergoes cyclic transformation from stages of rapid growth (anagen) to apoptosis-driven regression (catagen) via an interspersed period of relative quiescence (telogen). The cycling and regeneration of each HF depends on specialized mesenchymal dermal papilla cells and proliferating matrix cells located at the base of the follicle, and which are mediated by several molecules that control epithelial morphogenesis and growth. Several growth factors reportedly stimulate hair growth in *ex vivo* and animal models. For example, controlled release of vascular endothelial growth factor (VEGF) promotes the hair growth of the murine HF [1], and VEGF-mediated angiogenesis improves follicle vascularization and increases hair growth [2]. Platelet-derived growth factor (PDGF) isoforms reportedly induce and maintain the anagen phase of the murine HF and promote hair regeneration in mice [3]. Hepatocyte growth factor (HGF) and insulin-like growth factor (IGF) have also demonstrated the ability to up-regulate HF growth in various systems, such as, murine HF morphogenesis and cycling [4,5,6,7].

Keratinocyte stem/progenitor cells (KSCs) are known to reside in mostly two locations, the bulge region of the hair follicle and in non-random distributions within the basal layer of inter-follicular epithelium. KSCs, with a long-term proliferative possibility and a high short-term colony forming efficiency, express the integrin α_6_ strongly but weakly express a proliferation-associated cell surface marker, named transferrin receptor (CD71) (α_6_^bri^/CD71^dim^) [8,9]. KSCs continuously undergo self-renewal and generate transit-amplifying cells that rapidly divide to supply skin with new epithelial cells in the epidermal regeneration process. Moreover, KSCs play a critical role of in hair regeneration. For example, Kamimura *et al.* reported that HFs are composed of diverse cells of multiple origins and that the vast majority of reconstituted follicles appeared to be derived from KSCs [10]. In addition, Taylor *et al.* demonstrated that KSCs in a bulge give rise to HF cells as well as to upper follicular cells [11]. The KSC niche was defined by demonstrating that the bulge area contains the majority of infrequently cycling, label-retaining cells (*i.e.*, KSCs), which can respond to the anagen phase inducing signals from dermal papilla cells to regenerate hair follicle [12]. From previous study [13] and in this work, we defined high integrin α_6_ and low CD 71 (α_6_^bri^/CD71^dim^) positive keratinocyte cells as KSCs, as these subsets of keratinocytes have some characteristics of stem cells in the skin.

Secretion of growth factors and activation of neighboring cells are the origins of the mechanism of action of stem cells, and we have previously demonstrated that secretomes of adipose-derived stem cells promote hair growth in both HF organ culture and in animal studies [14]. Likewise, secretomes from KSCs may play a key role in hair regeneration via a paracrine mechanism in the HF. Therefore, in our present study we investigated: (1) whether KSC-conditioned medium (KSC-CM) promotes hair growth; and (2) which factor(s) secreted from KSCs mediate(s) hair-growth promotion. We studied the hair-growth promotion effects of KSC-CM on human HF organ culture and on a C3H/HeN mouse model. We then investigated the proliferative effect of KSC-CM on both human hair follicle dermal papilla cells (HFDPCs) and outer root sheath (ORS) cells.

## 2. Results

### 2.1. Effects of Keratinocyte Stem/Progenitor Cells Conditioned Medium (KSC-CM) on Hair Follicle Dermal Papilla Cells (HFDPCs)

**Figure 1:**
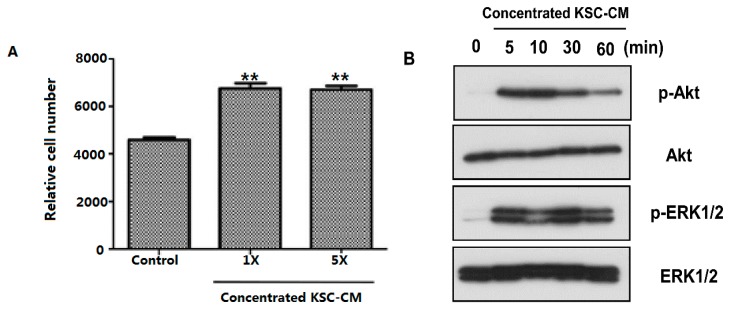

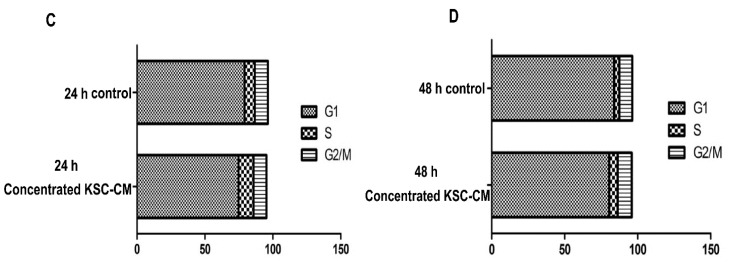
Effects of keratinocyte stem/progenitor cells conditioned medium (KSC-CM) on the proliferation of hair follicle dermal papilla cells (HFDPCs). (**A**) Cell proliferation was measured 48 h after concentrated KSC-CM treatment; (**B**) Western blot analysis against phospho-AKT and phospho-ERK1/2 showed that these pathways are activated by KSC-CM; (**C**,**D**) Cell cycle analysis of HFDPCs was performed by flow cytometry, and we found that the fraction of S phase cells was increased following 24 h (**C**) and 48 h (**D**) of concentrated KSC-CM treatment. ** *p* < 0.01.

Because hair cycle changes are influenced by rapid remodeling of both epithelial and dermal components [2], we investigated the proliferation of both HFDPCs and ORS cells. First, concentrated KSC-CM (1- and 5-fold) treatment significantly enhanced the proliferation of cultured HFDPCs (Figure 1A). To clarify the underlying mechanism of increased proliferation of HFDPCs, we analyzed the expression of signaling proteins related to cell proliferation using western blot analysis in HFDPCs. The level of phosphorylated AKT was significantly increased after concentrated (5-fold) KSC-CM treatment (Figure 1B). The expression of phosphorylated ERK1/2 was also increased with KSC-CM incubation (Figure 1B).

Because KSC-CM increased the proliferation of HFDPCs, cell cycle analysis of HFDPCs was performed in the presence of KSC-CM to determine whether the cell cycle was affected. Compared with the control group, incubation with concentrated KSC-CM for 24 h and 48 h decreased the length of the gap phase 1 (G_1_) of the cell cycle, while the proportion of the DNA synthesis phase (S) was increased (Figure 1C,D). This result indicates that KSC-CM has proliferative effects on HFDPCs by increasing the S phase fraction of the cell cycle.

### 2.2. Effects of KSC-CM on Outer Root Sheath Cells

**Figure 2:**
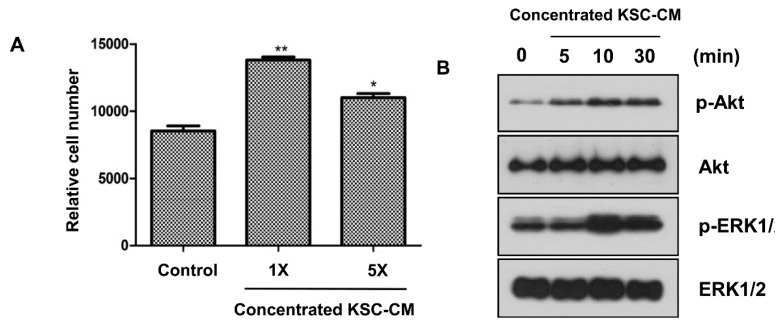
Effects of keratinocyte stem/progenitor cells conditioned medium (KSC-CM) on outer root sheath (ORS) cell proliferation. (**A**) Cell proliferation was measured 48 h after concentrated KSC-CM treatment; (**B**) Western blot analysis against phospho-AKT and phospho-ERK1/2 showed that these pathways are activated by KSC-CM. * *p* < 0.05; ** *p* < 0.01.

We investigated the proliferation of ORS cells and determined that treatment with 1- and 5-fold concentrated KSC-CM significantly enhanced the proliferation of cultured ORS cells (Figure 2A). The level of phosphorylated AKT was increased 5-fold after concentrated KSC-CM treatment (Figure 2B). In addition, the expression of phosphorylated ERK1/2 was significantly increased with KSC-CM incubation (Figure 2B). However, the cell cycle in KSC-CM treated ORS cells was not affected (data not shown).

### 2.3. Hair-Growth-Promoting Effects of KSC-CM in a C3H/HeN Mouse Model

**Figure 3:**
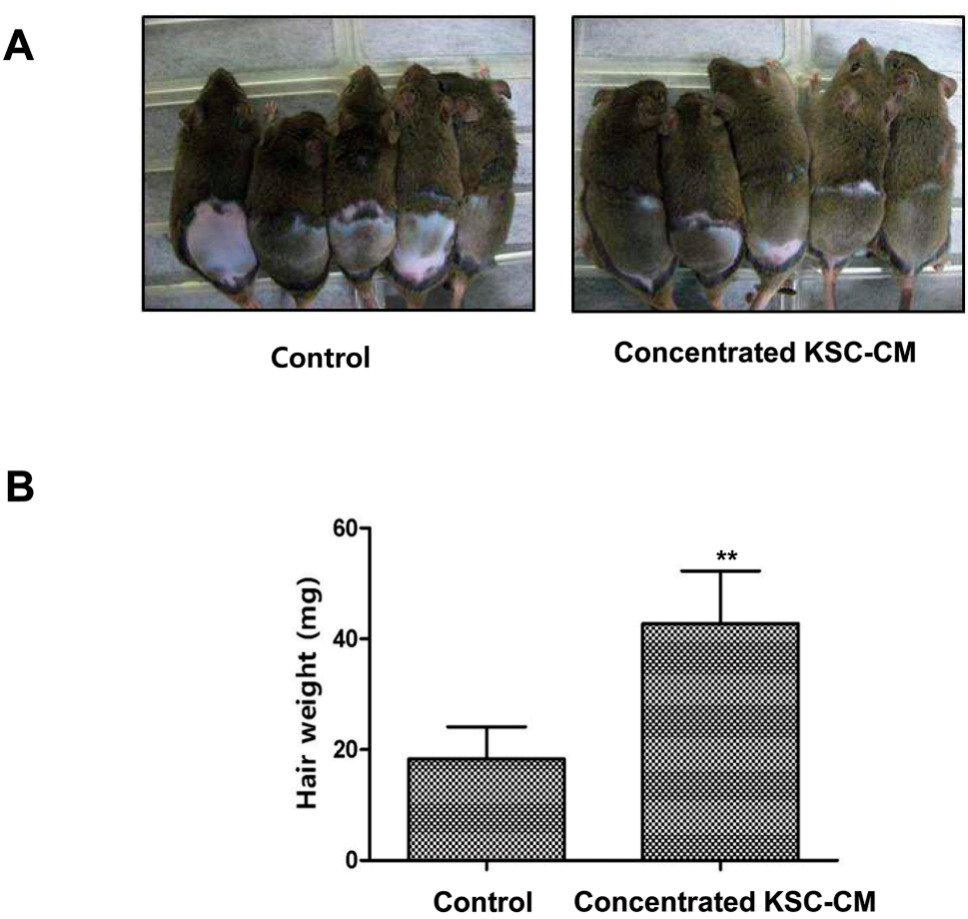
The promoting effects of keratinocyte stem/progenitor cells conditioned medium (KSC-CM) on hair growth in mice. Telogen-matched, seven-week-old C3H/NeH mice were shaved and subcutaneously injected three times with concentrated KSC-CM. (**A**) Photographs were taken two weeks after the injections; (**B**) The new hair was shaved, and the hair weight was measured. ** *p* < 0.01.

After topical injection of KSC-CM (Figure 3A) into the back of C3H/HeN mice, the conversion of telogen to anagen was induced earlier than in the controls (Figure 3A). The area close to the injection sites in the mice became darker in color after 10 days, thus indicating that HFs were in the anagen phase of the hair cycle. However, the injection sites of the controls retained their original white color. These findings indicate that locally injected KSC-CM might influence hair growth *in vivo*. In addition, the hair weight measured 14 days after concentrated KSC-CM treatment indicated that KSC-CM treatment significantly increased hair growth in the mice (Figure 3B). These findings indicate that KSC-CM can induce an earlier conversion of the hair cycle because the media stimulated hair growth in the murine model.

### 2.4. KSC-CM Increased Proliferation of Hair Matrix Keratinocytes Ex Vivo

**Figure 4:**
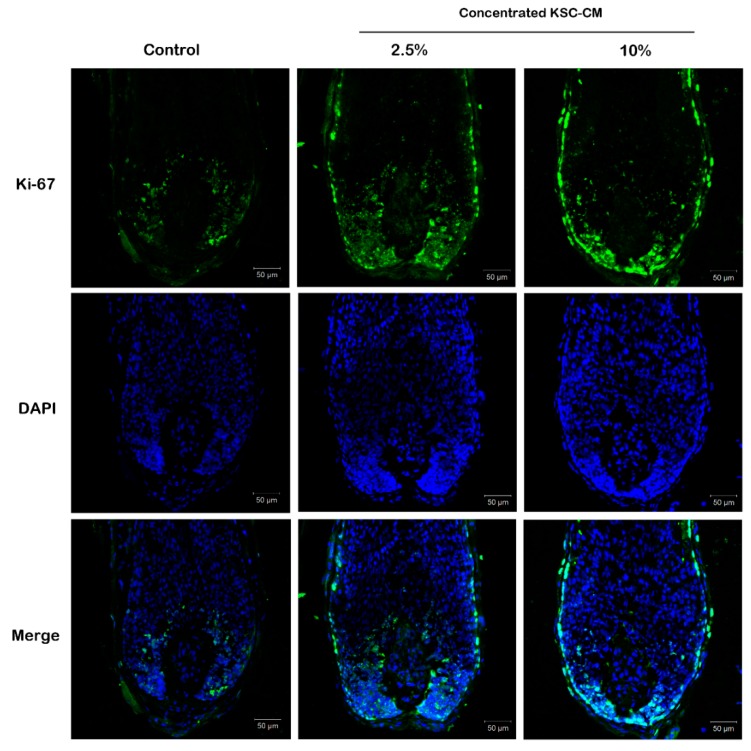
Effects of conditioned medium of keratinocyte stem/progenitor cells (KSC-CM) on *ex vivo* organ culture of human hair follicles. Isolated human anagen hairs were treated with KSC-CM (0%, 2.5% and 10%) in William’s E medium for two days. The Ki67-positive cells (green) were significantly increased by KSC-CM treatment; cells were counter-stained with 4',6-diamidino-2-phenylindole (DAPI).

The potential effect of KSC-CM on hair shaft elongation was investigated in isolated human anagen hairs. After the addition of KSC-CM (0%, 2.5% and 10%) to William’s E medium, Ki67-positive cells were stained green in *ex vivo* hair organ cultures. After two days of organ culture, the fluorescence intensity in the 2.5% KSC-CM-treated group increased by 40% compared with the control group treated with William’s E media alone (Figure 4). These results indicate that KSC-CM may stimulate hair growth by inducing the proliferation of follicular cells.

### 2.5. Identification of Proteins Involved in the Hair-Growth Promotion by KSC-CM

**Figure 5:**
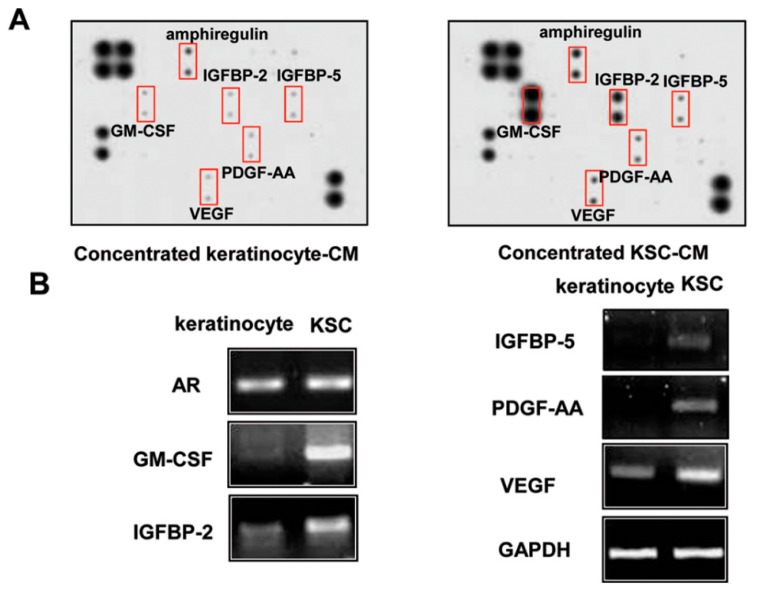
Altered protein secretion and mRNA expression in keratinocyte stem/progenitor cells (KSCs) and keratinocytes. (**A**) The growth-factor chip array showed that secretion of AREG, IGFBP2, IGFBP5, GM-CSF, PDGF-AA, and VEGF were significantly increased; (**B**) In addition, the mRNA expression of these growth factors is highly expressed in KSCs.

Because a paracrine effect is likely to be important for KSC-mediated hair regeneration, we examined the difference in secretion between keratinocytes and KSCs using a growth factor chip array [15,16,17,18]. The secretion of 41 growth factors was detected in conditioned medium obtained after a three-day culture with keratinocytes or KSCs. The secretion of amphiregulin (AREG), insulin-like growth factor binding protein-2 (IGFBP2), insulin-like growth factor binding protein-5 (IGFBP5), granulocyte macrophage-colony stimulating factor (GM-CSF), PDGF-AA, and VEGF was significantly increased in KSC-CM (Figure 5A). Consistent with the results seen in Figure 5A, RT-PCR analysis showed that KSCs showed significantly higher mRNA levels of *AREG*, *IGFBP2*, *IGFBP5*, *GM-CSF*, *PDGF-AA*, and *VEGF* than did keratinocytes (Figure 5B).

### 2.6. Hair-Growth-Promotion Effect of the Growth-Factor Complex

**Figure 6:**
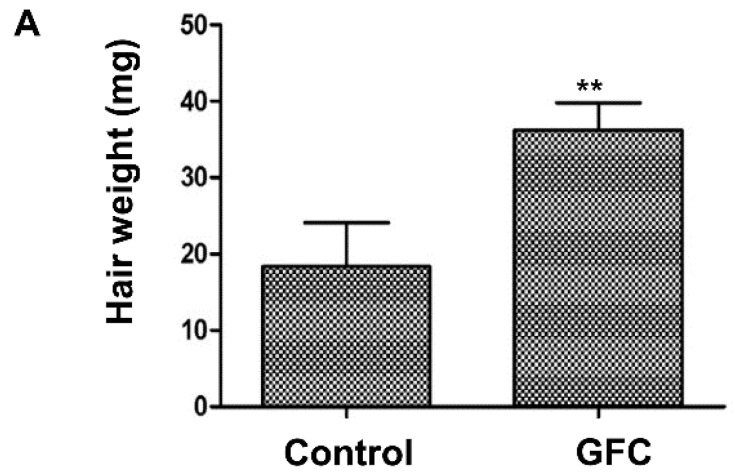

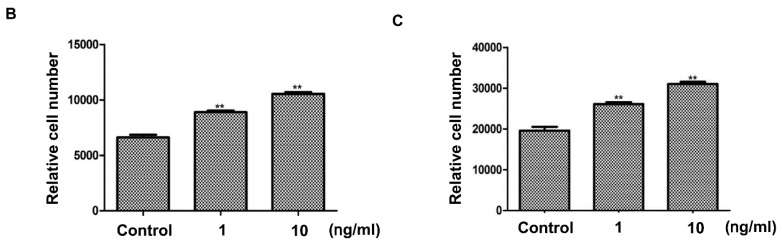
Effect of the growth-factor cocktail (GFC) on hair growth. (**A**) Seven-week-old C3H/NeH mice were shaved and subcutaneously injected three times with GFC. The hair weight was significantly increased by GFC treatment. In addition; GFC treatment significantly increased the proliferation of hair follicle dermal papilla cells (HFDPCs) (**B**) and outer root sheath (ORS) (**C**) cells. ** *p* < 0.01.

Because six growth factors are highly expressed in KSCs compared with keratinocytes, we investigated the hair-growth-promotion effect of the growth-factor cocktail GFC, which is a mixture of a 1 ng/mL concentration of these six recombinant growth factors. Figure 6A shows that GFC induced the conversion of the HF from telogen to anagen phase in C3H/HeN mice and also increased the hair weight (Figure 6A). In addition, GFC treatment (a mixture of a 1 ng/mL or 10 ng/mL concentration of the six recombinant growth factors) significantly induced the proliferation of HFDPCs (Figure 6B) and of ORS cells (Figure 6C). Collectively, these results indicate that these growth factors might be responsible for the hair-growth-promotion effect of KSC-CM.

## 3. Discussion

Because KSCs may have a key role in hair regeneration via a paracrine mechanism, in the present study we investigated whether KSC-CM could promote hair growth as well as which factors in KSC-CM might mediate this process. We found that concentrated KSC-CM significantly increased the proliferation of HFDPCs and ORS cells, in part, through the phosphorylation of AKT and ERK1/2. Concentrated KSC-CM treatment accelerated hair growth in C3H/HeN mice and increased the proliferative fraction of Ki67-positive hair matrix keratinocytes in human HFs. We identified six secreted growth factors that were enriched in KSC-CM and found that a cocktail of these proteins (1 ng/mL concentration of each protein) accelerated the hair growth in a mouse model as well as HFDPCs and ORS cells.

In a preliminary study, we co-cultured keratinocytes and HFDPC cells in a trans-well system, but found that keratinocytes did not increase the proliferation of DPCs (data not shown). Therefore, we identified the six growth factors by comparing the secretomes of KSCs and keratinocytes using a growth-factor chip array. KSC-CM contains proliferative stimulators including angiogenic factors such as AREG, IGFBP2, IGFBP5, GM-CSF, PDGF-AA, and VEGF. We found that the combination of these factors stimulates hair growth. RT-PCR analysis also confirmed that KSCs express high basal levels of *AREG*, *IGFBP2*, *IGFBP6*, *GM-CSF*, *PDGF-AA*, and *VEGF* mRNAs compared with keratinocytes (Figure 5B). However, it is unlikely that only these factors are involved in the hair growth of KSCs. Because of the limitations of the assay system we used in this study, we could measure only 41 growth factors in the conditioned medium. Therefore, there may be additional factors which accelerate the hair growth in KSC-CM. Further studies will be needed to identify the key paracrine factors. We also tested whether the addition of a single growth factor could increase the proliferation of HFDPCs, and found that no single factor significantly increased the proliferation in our preliminary study. Therefore, it is reasonable to conclude that the growth factors in KSC-CM collectively promote hair growth.

The positive roles of growth factors on hair-growth stimulation are well-known. IGF-1 is essential for normal hair growth and development, and may also be important in regulating the hair-growth cycle [19]. In many culture systems IGF-1 actions are modulated by the IGFBPs, which are also reportedly involved in controlling hair growth [7,20]. In mice, PDGF isoforms markedly promote hair regeneration by regulating the anagen phase of the murine HF [3]. Controlled release of VEGF increases hair growth in the murine HF [1], and VEGF-mediated angiogenesis also improves follicle vascularization as well [2]. However, the functional roles of AREG and GM-CSF on hair growth have not yet been reported.

## 4. Experimental Section

### 4.1. Cell Cultures

HFDPCs were obtained from PromoCell (Heidelberg, Germany) and grown in Follicle Dermal Papilla Cell Growth Medium with Supplement Mix (PromoCell) at 37 °C in 5% CO_2_. ORS cells and keratinocytes were cultured as previously described [21], and were grown in EpiLife (GIBCO, Invitrogen, Carlsbad, CA, USA) at 37 °C in 5% CO_2_. KSCs were obtained from CELLnTEC (Bern, Switzerland) and were grown in Keratinocyte Growth Medium 2 with Supplement Mix (PromoCell) at 37 °C in 5% CO_2_. KSCs were characterized by flow cytometry (FACS) analysis using antibodies to integrin α6 and CD 71 as described in a previous study [13].

### 4.2. Preparation and Concentration of KSC-CM

Concentrated KSC-CM was prepared according to the method of Kim *et al.* [22,23], although with some modifications. Briefly, KSCs became 100% confluent, and the medium was replaced with serum-free medium. After 72 h, conditioned medium was collected, centrifuged at 1800 rpm for 10 min, and filtered through a 0.22-μm syringe filter. The filtrate was then centrifuged in 3 kDa molecular-weight-cut-off Vivaspin (Sartorius Stedim Biotech GmbH, Goettingen, Germany) and concentrated.

### 4.3. Proliferation Assay

HFDPCs or ORS cells were seeded at a density of 5000 cells/cm^2^ in 48-well plates. After 24 h of incubation, the culture medium was replaced by new medium containing various concentrations of concentrated KSC-CM, and the cells were then incubated for 72 h in order to proliferate. A cell proliferation assay was then performed using a Cell-Counting Kit-8 (CCK-8, Dojindo Laboratories, Kumamoto, Japan). CCK-8 solution (150 μL) was added, and each well was incubated for 2 h. Following incubation, the absorbance was measured at 450 nm using an enzyme-linked immunosorbent assay (ELISA) reader (TECAN, Grodig, Austria).

### 4.4. FACS Analysis

FACS analysis was performed as previously described [22]. After serum starvation for 24 h, cells were incubated using supplement-free media or concentrated KSC-CM. After 48 h, cells were then harvested, washed twice with PBS, permeabilized with 70% ethanol, and finally stained with 0.2 mg/mL RNase A and 50 μg/mL propidium iodide. The cells were analyzed for the DNA cycle using a flow cytometer (Becton-Dickinson, San Jose, CA, USA).

### 4.5. Western Blotting

Protein samples were prepared in RIPA buffer (50 mM Tris-HCl (pH 7.4), 150 mM NaCl, 1 mM EDTA, 1% Triton-X 100, 1% SDS, 50 mM NaF, 1 mM Na_3_VO_4_, 5 mM dithiothreitol, 1 mg/mL leupeptin, and 1 mM PMSF). Samples were separated on 12% SDS-polyacrylamide gel, transferred to PVDF membranes, and blocked with 5% dried milk in PBS containing 0.4% Tween-20. The blots were incubated with the appropriate primary antibodies that recognize AKT, phospho-specific AKT (Ser473; Cell Signaling, Danvers, MA, USA), ERK1/2 (Cell Signaling), phospho-specific ERK1/2 (Cell Signaling), and actin (Santa Cruz Biotechnology, Inc., Santa Cruz, CA, USA). Membrane-bound primary antibodies were detected using secondary antibodies conjugated with horseradish peroxidase and Immobilon™ western chemiluminescent HRP reagent (Millipore, Bedford, MA, USA) and exposed to X-ray film (Agfa, Mortsel, Belgium).

### 4.6. Animal Studies

Protocols for the animal studies were approved by the Institutional Animal Care and Use Committee of the Seoul National University (Seoul, Korea). Female C3H/HeN mice (6 weeks old and weighing 20–25 g) were purchased from Orient Bio, Inc. (Seongnam, Korea). The mice were kept in a clean room (Animal Center for Pharmaceutical Research, College of Pharmacy, Seoul National University) at a temperature between 20–23 °C, with 12 h light (07:00–19:00) and dark (19:00–07:00) cycles, and with a relative humidity of 50% ± 5%. The mice were housed in metabolic cages (Tecniplast, Varese, Italy) in filtered, pathogen-free air and with both food (Agribrands Purina, Pyeongtaek, Korea) and water available *ad libitum*. Hair growth studies were conducted in seven-week-old C3H/HeN mice; HFs were synchronously matched in the telogen stage. The dorsal side of each mouse was shaved using a clipper and electric shaver with special care taken in order to avoid damaging the bare skin. Three subcutaneous injections of 0.1% BSA-PBS (control), growth-factor cocktail (GFC), and concentrated KSC-CM were then made in the dorsal skin of each mouse at two-day intervals, and any darkening of the skin was monitored. After two weeks, the re-grown dorsal hair was shaved and its weight was measured.

### 4.7. Ex Vivo Human HF Organ Culture Study

Human occipital scalp HFs were isolated from volunteers who had given informed consent, and the HFs were cultured *in vitro*, as described previously [24]. Briefly, dissected HFs were cut into small pieces, approximately 2 mm in length from the bottom of the dermal papilla, and were cultured in William’s E medium (Gibco BRL, Gaithersburg, MD, USA) with 10 ng/mL hydrocortisone, 10 ng/mL insulin, 2 mM l-glutamine, and 100 U/mL penicillin at 37 °C in a 5% CO_2_ atmosphere. Anagen HFs were cultured from three different volunteers. KSC-CM (0%, 2.5% and 10%) was added to the basal William’s E medium. HFs cultured in William’s E media were used for a negative control. After culturing for two days, the HF organs were then harvested and were ultimately stained with anti-Ki-67 (1:100; Becton Dickinson, Franklin Lakes, NJ, USA) and DAPI.

### 4.8. Human Growth Factor Chip Array

A human growth factor chip array kit was purchased from R&D systems (Minneapolis, MN, USA). Briefly, membranes were placed in an 8-well tissue culture tray and were incubated with 2 mL of 1× blocking buffer at room temperature for 1 h. The membrane was then incubated overnight at 4 °C with 1 mL of concentrated control or concentrated KSC-CM (total 200 μg of protein). After decanting the samples, 1 mL of diluted detection antibody cocktail A was incubated with a membrane for 2 h at room temperature. After incubation with diluted streptavidin-HRP at room temperature for 30 min, a signal was detected using a chemiluminescent substrate system (Immobilon western reagent), and the signal was quantified using a densitometer (Bio-Rad Laboratories, Hercules, CA, USA). The background intensity was subtracted for the analysis. The data were normalized to the positive control values expressed in the membrane.

### 4.9. Reverse Transcriptase-Polymerase Chain Reaction (RT-PCR) Analysis

Total RNA was extracted using TRIzol reagent (Invitrogen) followed by reverse transcription using a cDNA synthesis kit (Promega, Madison, WI, USA). cDNA was synthesized from 1 µg of total RNA using 200 U of reverse transcriptase and 20 pM oligo-dT. The following oligonucleotides were used as primers: *VEGF* (5'-TAC CTC CAC CAT GCC AAG T-3' and 5'-TGC ATT CAC ATT TGT TGT GC-3'); *AREG* (5'-AAG CGT GAA CCA TTT TCT GG-3' and 5'-AGC CAG GTA TTT GTG GTT CG-3'); *GM-CSF* (5'- ACT GCT GCT GAG ATG AAT GA-3' and 5'-AGG GCA GTG CTG CTT GTA GT-3'); *IGFBP-2* (5'-CCC TCA AGT CGG GTA TGA AG-3' and 5'-ACC TGG TCC AGT TCC TGT TG-3'); *IGFBP-5* (5'-GAA TCC AGG CAC CTC TAC CA-3' and 5'-GGT AGA AGC CTC GAT GGT CA-3'); *PDGF-AA* (5'-CAA GAC CAG GAC GGT CAT TT-3' and 5'-CCT GAC GTA TTC CAC CTT GG-3'); and the internal control *GADPH* (5'-CGA GAT CCC TCC AAA ATC AA-3' and 5'-TGT GGT CAT GAG TCC TTC CA-3'). PCRs were performed in a final volume of 20 µL of reaction mix that contained 2 µL of the RT reaction mixture, 15 mM MgCl_2_, 1.25 mM dNTPs, 20 pM of each primer, and 0.5 U of Taq polymerase (Promega). Thermal cycling consisted of an initial denaturation at 94 °C for 5 min, amplification for 35 cycles (94 °C for 30 s, 56 °C for 30 s, and 72 °C for 30 s), and termination by a final extension at 72 °C for 5 min. The *GAPDH* mRNA level was used for sample standardization. After electrophoresis on 1.5% agarose gel, each band was quantified using a densitometer (Bio-Rad Laboratories).

### 4.10. Statistics

Differences among treatments were assessed by an analysis of variance (ANOVA) and followed by Dunnett’s test. *p*-values of <0.05 were regarded as significant.

## 5. Conclusions

In this study, we have found that high integrin α_6_ and low CD 71 (α_6_^bri^/CD71^dim^) positive keratinocyte cells increases the proliferation of HFDPCs and ORS cells. High integrin α_6_ and low CD 71 (α_6_^bri^/CD71^dim^) positive keratinocyte cells treatment accelerates hair growth in C3H/HeN mice and increases the number of Ki67-positive hair matrix keratinocytes. Thus, high integrin α_6_ and low CD 71 (α_6_^bri^/CD71^dim^) positive keratinocyte cells or a mixture of recombinant growth factors can be used for hair-growth stimulation.

## References

[1] Ozeki M., Tabata Y. (2002). Promoted growth of murine hair follicles through controlled release of vascular endothelial growth factor. Biomaterials.

[2] Yano K., Brown L.F., Detmar M. (2001). Control of hair growth and follicle size by VEGF-mediated angiogenesis. J. Clin. Investig..

[3] Tomita Y., Akiyama M., Shimizu H. (2006). PDGF isoforms induce and maintain anagen phase of murine hair follicles. J. Dermatol. Sci..

[4] Jindo T., Tsuboi R., Takamori K., Ogawa H. (1998). Local injection of hepatocyte growth factor/scatter factor (HGF/SF) alters cyclic growth of murine hair follicles. J. Investig. Dermatol..

[5] Lindner G., Menrad A., Gherardi E., Merlino G., Welker P., Handjiski B., Roloff B., Paus R. (2000). Involvement of hepatocyte growth factor/scatter factor and met receptor signaling in hair follicle morphogenesis and cycling. FASEB J..

[6] Su H.Y., Hickford J.G., Bickerstaffe R., Palmer B.R. (1999). Insulin-like growth factor 1 and hair growth. Dermatol. Online J..

[7] Weger N., Schlake T. (2005). IGF-I signalling controls the hair growth cycle and the differentiation of hair shafts. J. Investig. Dermatol..

[8] Kaur P. (2006). Interfollicular epidermal stem cells: Identification, challenges, potential. J. Investig. Dermatol..

[9] Webb A., Li A., Kaur P. (2004). Location and phenotype of human adult keratinocyte stem cells of the skin. Differentiation.

[10] Kamimura J., Lee D., Baden H.P., Brissette J., Dotto G.P. (1997). Primary mouse keratinocyte cultures contain hair follicle progenitor cells with multiple differentiation potential. J. Investing. Dermatol..

[11] Taylor G., Lehrer M.S., Jensen P.J., Sun T.T., Lavker R.M. (2000). Involvement of follicular stem cells in forming not only the follicle but also the epidermis. Cell.

[12] Tumbar T., Guasch G., Greco V., Blanpain C., Lowry W.E., Rendl M., Fuchs E. (2004). Defining the epithelial stem cell niche in skin. Science.

[13] Sung S.H., Park S.H., Song S.Y., Lee S.J., Lee H.W., Kim S.H., A Lee M., Yoon I.S., Kim D.D., Kang S. (2011). Epidermal regeneration by ENT-16α, 17-dihydroxy-kauran-19-oic acid isolated from *Siegesbeckia pubescens*. Cell Prolif..

[14] Won C.H., Yoo H.G., Kwon O.S., Sung M.Y., Kang Y.J., Chung J.H., Park B.S., Sung J.H., Kim W.S., Kim K.H. (2010). Hair growth promoting effects of adipose tissue-derived stem cells. J. Dermatol. Sci..

[15] Kim W.S., Park B.S., Sung J.H. (2009). Protective role of adipose-derived stem cells and their soluble factors in photoaging. Arch. Dermatol. Res..

[16] Park B.S., Jang K.A., Sung J.H., Park J.S., Kwon Y.H., Kim K.J., Kim W.S. (2008). Adipose-derived stem cells and their secretory factors as a promising therapy for skin aging. Dermatol. Surg..

[17] Song S.Y., Chung H.M., Sung J.H. (2010). The pivotal role of VEGF in adipose-derived-stem-cell-mediated regeneration. Expert Opin. Biol. Ther..

[18] Lee E.Y., Xia Y., Kim W.S., Kim M.H., Kim T.H., Kim K.J., Park B.S., Sung J.H. (2009). Hypoxia-enhanced wound-healing function of adipose-derived stem cells: Increase in stem cell proliferation and up-regulation of VEGF and bFGF. Wound Repair Regen..

[19] Batch J.A., Mercuri F.A., Werther G.A. (1996). Identification and localization of insulin-like growth factor-binding protein (IGFBP) messenger RNAs in human hair follicle dermal papilla. J. Investig. Dermatol..

[20] Park B.S., Kim W.S., Choi J.S., Kim H.K., Won J.H., Ohkubo F., Fukuoka H. (2010). Hair growth stimulated by conditioned medium of adipose-derived stem cells is enhanced by hypoxia: Evidence of increased growth factor secretion. Biomed. Res..

[21] Kwack M.H., Shin S.H., Kim S.R., Im S.U., Han I.S., Kim M.K., Kim J.C., Sung Y.K. (2009). l-Ascorbic acid 2-phosphate promotes elongation of hair shafts via the secretion of insulin-like growth factor-1 from dermal papilla cells through phosphatidylinositol 3-kinase. Br. J. Dermatol..

[22] Kim W.S., Park B.S., Park S.H., Kim H.K., Sung J.H. (2009). Antiwrinkle effect of adipose-derived stem cell: Activation of dermal fibroblast by secretory factors. J. Dermatol. Sci..

[23] Kim W.S., Park B.S., Kim H.K., Park J.S., Kim K.J., Choi J.S., Chung S.J., Kim D.D., Sung J.H. (2008). Evidence supporting antioxidant action of adipose-derived stem cells: Protection of human dermal fibroblasts from oxidative stress. J. Dermatol. Sci..

[24] Krugluger W., Rohrbacher W., Laciak K., Moser K., Moser C., Hugeneck J. (2005). Reorganization of hair follicles in human skin organ culture induced by cultured human follicle-derived cells. Exp. Dermatol..

